# Binge drinking and total alcohol consumption from 16 to 43 years of age are associated with elevated fasting plasma glucose in women: results from the northern Swedish cohort study

**DOI:** 10.1186/s12889-017-4437-y

**Published:** 2017-06-08

**Authors:** Karina Nygren, Anne Hammarström, Olov Rolandsson

**Affiliations:** 1 Department of Public Health and Clinical Medicine, Umeå University, Umeå, SE 90187 Sweden; 2 Department of Public Health and Caring Sciences, Uppsala University, Uppsala, Sweden

**Keywords:** Glucose metabolism, Alcohol, Gender, Cohort study

## Abstract

**Background:**

Studies have indicated that moderate alcohol consumption is associated with lower incidence of diabetes in women. However, not only the amount but also the drinking pattern could be of importance when assessing the longitudinal relation between alcohol and glucose. Also, there is a lack of studies on alcohol use beginning in adolescence on adult glucose levels. The aim was to examine the association between total alcohol consumption and binge drinking between ages 16 and 43 and fasting plasma glucose at age 43.

**Methods:**

Data were retrieved from a 27-year prospective cohort study, the Northern Swedish Cohort. In 1981, all 9th grade students (*n* = 1083) within a municipality in Sweden were invited to participate. There were re-assessments at ages 18, 21, 30 and 43. This particular study sample consisted of 897 participants (82.8%). Fasting plasma glucose (mmol/L) was measured at a health examination at age 43. Total alcohol consumption (in grams) and binge drinking were calculated from alcohol consumption data obtained from questionnaires.

**Results:**

Descriptive analyses showed that men had higher levels of fasting plasma glucose as compared to women. Men also reported higher levels of alcohol consumption and binge drinking behavior. Linear regressions showed that total alcohol consumption in combination with binge drinking between ages 16 and 43 was associated with elevated fasting plasma glucose at age 43 in women (beta = 0.14, *p* = 0.003) but not in men after adjustment for BMI, hypertension and smoking at age 43.

**Conclusions:**

Our findings indicate that reducing binge drinking and alcohol consumption among young and middle-aged women with the highest consumption might be metabolically favorable for their future glucose metabolism.

## Background

Several studies have indicated that moderate alcohol consumption is associated with reduced risk for type 2 diabetes [1–3], and that women seem to benefit more from moderate alcohol consumption than men [1]. The association has been reported to be J-shaped i.e. low to moderate consumers have a lower risk of diabetes compared to both abstainers but also high alcohol consumers [4] The J-shaped association has been taken as proof of one of the benefits of alcohol. However, as suggested by the J-shaped association, high alcohol consumers are at high risk of developing type 2 diabetes or the metabolic syndrome [5]. This was found in e.g. the National Health and Nutrition Examination Survey where persons with high alcohol consumption or who engaged in binge drinking behavior had an increased risk of the metabolic syndrome [6], a finding which was corroborated in a Swedish cohort study [7]. The Swedish cohort study also showed that high alcohol consumption and binge drinking increased the risk of type 2 diabetes as well as pre-diabetes for men, but not for women [7].Also, a recent meta-analysis of prospective studies suggested that heavy drinking was associated with the metabolic syndrome that includes an increase in blood glucose [8].

The majority of the studies on the association between alcohol consumption and glucose have been performed in adults. Little is known about the effect of alcohol consumption as an adolescent on glucose concentrations in adult life. However, in a longitudinal study who followed a group of adolescents (mean age 15.7) until they were young adults (mean age 28.7) reported an increased risk of diabetes in those who consumed alcohol 3–7 days per week [9].

Taken together, there is a lack of longitudinal studies studying the association between alcohol consumption and blood glucose following adolescents until adulthood. Thus, our aim was to examine the association between total alcohol consumption and binge drinking from 16 to 43 years of age and fasting plasma glucose measured at age 43.

## Methods

Data were based on the prospective longitudinal cohort study called the Northern Swedish Cohort [10]. In 1981, all pupils (*n* = 1083) aged 16 years who attended or should have attended the final year of the compulsory school in a middle-sized municipality in northern Sweden participated in the study. There have been four follow-up surveys at ages 18, 21, 30, and 43. At age 16, the participation rate was 99.7% (1080/1083). In the final follow-up, 94.3% (1010/1071) of those still alive (12 persons had passed away) participated in the study.

The study sample used in the present study consisted of 897 persons (433 women, 464 men); participants who did not have data on fasting plasma glucose (*n* = 86). as Also, participants who at any follow-up reported having type 1 diabetes (*n* = 10) or type 2 diabetes (*n* = 17) were excluded due to a risk of their fPG being affected as a consequence of their disease. Type 2 diabetes is a complex disease affecting many organs that are involved in glucose regulation; thus, having a type 2 diagnose might confound the relationship between fPG per se and alcohol consumption. Secondly, they filled in the questionnaire after their diagnosis, which increased the risk of recall bias. Finally, they were too few to perform sub-group analysis on. Body Mass Index (BMI) and hypertension at age 43 was higher for cohort participants who were not included for this study (mean BMI = 29.6 ± 8.3, hypertension 45.6%) than for the study sample (mean BMI =26.7 ± 4.7, *p*-value = 0.02, hypertension 24.8%, *p*-value = <0.001). No significant differences were observed between the two groups regarding alcohol consumption.

Ethical approvals were given by the Regional Ethics Review Boards in Uppsala and Umeå, Sweden. According to Swedish law (Swedish Ethical Review Act 2003; 460, §17), written consents are not required in a questionnaire study such as ours. Instead, each respondent is viewed as giving his/her written consent when answering the questionnaire. The participants of the study were informed of their right to opt out at any time simply by not completing any wave(s) of the survey.

### Measurements

The participants underwent a clinical health examination at age 43. A blood sample was drawn after an overnight fast, and fasting plasma glucose (fPG) was analyzed (Vitros, 5.1 FS, Ortho-Clinical Diagnostics J&J, Raritan, NJ). External quality assurance in laboratory medicine in Sweden (EQUALIS) managed external quality control.

Weight (kg) and height (m) were measured at age 16 and 43 according to the World Health Organization Multinational Monitoring of Trends and Determinants in Cardiovascular Disease (WHO MONICA) manual [11], and BMI was calculated (kg/m^2^). BMI was also dichotomized into normal weight (BMI <25) and overweight/obese (BMI ≥25) [12]. Health examinations at age 43 also included measures of participants’ waist circumference and blood pressure, measured according to WHO [11]. Participants were coded as 1 = hypertensive (a blood pressure level ≥ 140/90 mmHg or being treated with anti-hypertensive medication) or 0 = normotensive.

### Questionnaire data

The participants responded to a questionnaire at each survey regarding issues such as their health status, lifestyle, and socio-economic conditions.

*Alcohol consumption.* The participants responded to eight ordinal-scale questions concerning their consumption of medium-strength beer, strong beer, wine, and spirits. The participants were asked questions concerning frequency, ‘How often do you drink beer?’, and volume ‘How much beer do you usually drink at each occasion?’. Standard drink units according to Swedish National Institute of Public Health standards were used [13–15]. One standard drink contained 12 g of ethanol, which in turn equaled 4 cl spirits (40% pure ethanol), 12–15 cl wine (11–13% pure ethanol), 33 cl strong beer (5–6% pure ethanol), or 50 cl medium-strength beer (2–3% pure ethanol).

**Fig. 1:**
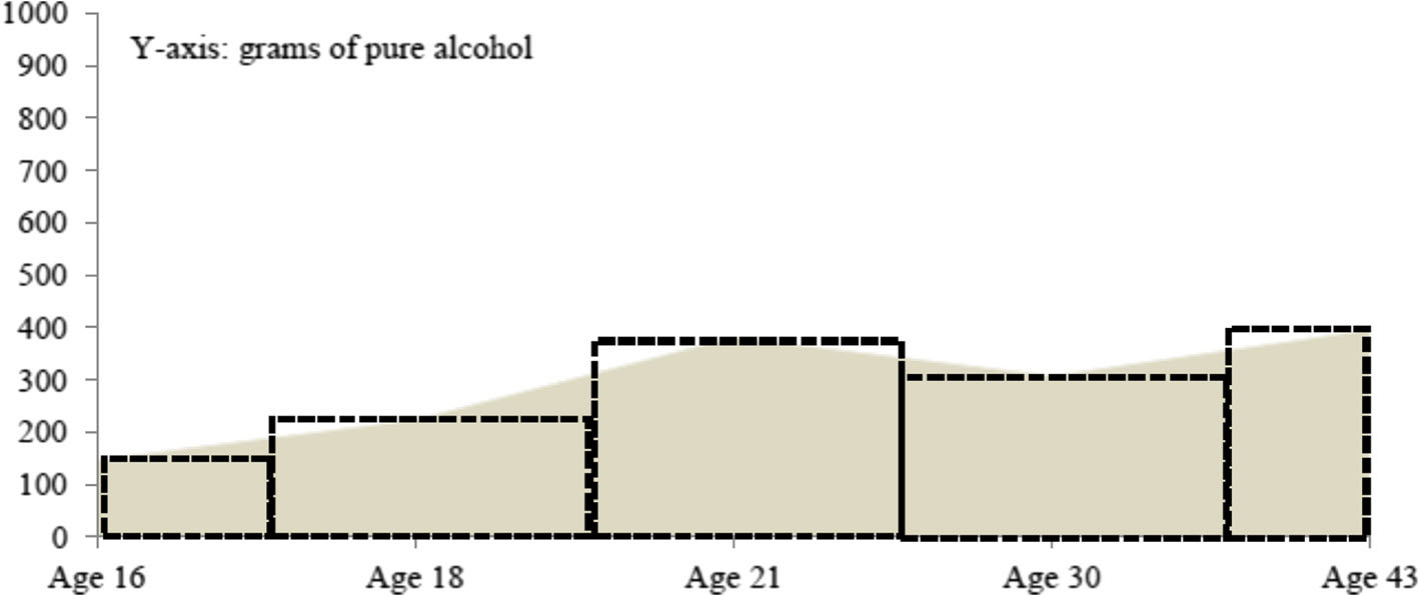
Calculation of total alcohol consumption ages 16–43. Value of alcohol consumption ages 16, 18, 21, 30 and 43 respectively treated as mean values and multiplied by number of years (no of years between the prior follow-up divided by two + no of years between the between each survey wave (black boxes)

*The total alcohol consumption between ages 16 and 43* was calculated in a step-wise manner. Frequency (occasions per year) and volume (grams of alcohol per occasion) variables were created, after which they were multiplied for each type of alcohol and the totals for each type of alcohol were summed. To calculate the alcohol consumption for the years in between the follow-ups, an area under the curve (trapezium rule) was calculated (see Fig. 1) [16]. Alcohol consumption at ages 16, 18, 21, 30, and 43 were assumed to be representative (mean values) of longer time-spans. The number of years between each follow-up was divided in two, where the first half was credited to the former follow-up and the second half was credited to the latter follow-up. The alcohol consumption mean values were multiplied by years as follows: the alcohol consumption level at age 16 was multiplied by 1 (age 16 to 17); the alcohol consumption level at age 18 was multiplied by 2.5 (age 17 to 19.5). At age 21, the alcohol consumption level was multiplied by 6 (age 19.5 to 25.5). The alcohol consumption level at age 30 was multiplied by 11.5 (age 25.5 to 37), and the alcohol consumption level at age 43 was multiplied by 7 (from age 37 to and including age 43). The consumption measures for each time-span were then added to a measure (grams of pure alcohol) of total alcohol consumption from 16 years of age to 43 years of age.

In addition, total alcohol consumption variables were created for each type of alcohol (beer, wine and spirits). Total alcohol consumption ages 16 to 43 was also divided into quintiles for descriptive purposes.

*Binge drinking between ages 16 and 43* was also calculated in several steps. First, binge drinking was defined as drinking 4 or more standard drinks of beer, wine or spirits per occasion for women (5 or more standard drinks for men) at least once a month [17, 18]. This variable was coded as 0 = ‘not binge drinking’ and 1 = ‘binge drinking’. Second, a composite measure of binge drinking between ages 16–43 was created, in which time between data collection points were accounted for. Each dichotomous binge drinking measures was multiplied with a certain number of years, estimated by the data collection closest in time; in other words, the multiplicative procedure was the same as in the total alcohol consumption variable (binge drinking at age 16*1 year, binge drinking at age 18*2.5 years, binge drinking at age 21*6 years, binge drinking at age 30*11.5 years, and binge drinking at age 43*7 years), creating an index ranging from 0 to 28. Binge drinking between ages 16 and 43 was thus defined as number of years during which the respondent reported binge drinking.

*A combined total alcohol consumption and binge drinking variable (ages 16 to 43)* was also created in order to examine the combined effect of the two alcohol variables. The total alcohol consumption variable between ages 16 and 43 was dichotomized at the 75th percentile (cut-offs were 33,155 g for women and 95,536 g for men), and coded as 0 = ‘total alcohol consumption below 75^th^ percentile’ and 1 = ‘total alcohol consumption above 75^th^ percentile’ [19]. The binge drinking between ages 16 and 43 variable was dichotomized and coded as 0 = ‘no binge drinking’ and 1 = ‘between one and twenty-eight years of binge drinking’. The two dichotomized variables were then combined and coded as 0 = ‘no binge drinking and total alcohol consumption less than 75^th^ percentile’, 1 = ‘either between one and twenty-eight years of binge drinking or total alcohol consumption over 75^th^ percentile’, 2 = ‘between one and twenty-eight years of binge-drinking and total alcohol consumption over 75^th^ percentile’.

*Family history of diabetes and working-class parents at age 16.* At age 16, the participants indicated whether any of their parents or siblings had diabetes. Participants’ working-class background was measured with an open-ended question concerning the current or previous occupations of their parents. The responses were classified according to a Swedish classification system [20] and added into a combined variable coded as 0 = ‘no working-class parent’, 1 = ‘one working-class parent’, or 2 = ‘two working-class parents’.

*Smoking, intake of sugary foods, and exercise at age 16 and 43.* These covariates were based on ordinal scale level questionnaire items. Smoking ranged from 0 = ‘non-smoker’ to 4 = ‘smokes more than 20 cigarettes per day’. Intake of sugary foods ranged from 0 = ‘more seldom than once a week’ to 4 = ‘several times a day’. Finally, exercise habits ranged from 0 = ‘daily’ to 5 = ‘seldom/never’.

All covariates were also dichotomized for descriptive purposes.

### Statistical analysis

Data are presented as number (n), proportions (%), median values (interquartile range, IQR) and mean values (standard deviations, SD). All analyses were performed separately for men and women. Differences between two groups were tested with Chi-squared tests, Mann–Whitney U test, or Student’s *t*-test. Differences between more than two groups were tested through Analysis of Variance (ANOVA). ANOVA analyses also included a test for linear trend of the mean fPG values between the groups.

In preliminary analyses of the relationship between fPG and total alcohol consumption between ages 16 and 43, we also performed scatter plots, residual plots, and tests for a quadratic trend. The quadratic trend was examined by performing multivariable linear regressions with a mean centered total alcohol consumption variable and a quadratic mean centered total alcohol consumption variable as independent variables.

Univariate and multivariable linear regression were used to examine the associations between alcohol consumption with fPG as the dependent variable. In the multivariable analysis, we included the independent variables that were significantly associated with fPG in the univariate analysis; i.e. all non-significant covariates were excluded. Due to strong correlations between the alcohol consumption variables, three separate multivariable models were presented. Collinearity was found between BMI at ages 16 and 43, resulting in the exclusion of BMI at age 16 from the multivariable analysis.

Due to significant interaction between gender and BMI at age 43 (beta 0.71, *p* = 0.002), a subsequent linear regression analysis was performed in which we stratified for BMI (BMI <25 and BMI ≥25) at age 43. In all other analyses, BMI was used as a continuous variable. All multivariable linear regressions included a test of collinearity by estimation of variance inflation factor.

We performed a sensitivity analysis in which we excluded five total alcohol consumption outliers. These were participants (four men, one woman) who reported an alcohol consumption of more than 800,000 g of pure alcohol, significantly higher as compared to other study participants. A second sensitivity analysis was performed in which participants who had never reported drinking alcohol were excluded.

The significance level for all analyses was *p < 0.05 (two-sided). All analyses were performed using IBM-SPSS version 21.

## Results

Men had higher fPG compared to women at age 43 and had consumed almost three times as much alcohol as women from 16 to 43 years of age (Table 1). In addition, a larger proportion of men reported binge drinking. There were more men than women who were overweight and obese at age 43 but not at age 16, and more men reported exercising a few times a month or more seldom at age 43. However, more women were daily smokers compared to men at age 16 and 43 (Table 1).

**Table 1:** Characteristics of the study population by gender at age 16 and 43

		Women (*n* = 433)	Men (*n* = 464)	*p*-value
Fasting plasma glucose				
Fasting plasma glucose at age 43 (mmol/L)		4.9 (0.6)	5.3 (1.0)	<0.001
Alcohol consumption				
Total alcohol consumption from ages 16 to 43 (grams of pure alcohol)		17,995 (22216)	48,864 (66323)	<0.001
Binge drinking from ages 16 to 43^a^, range 0–28 (years)		0.0 (9.50)	11.5 (16.88)	<0.001
Combined total alcohol consumption and binge drinking from ages 16 to 43^b^, range 0–2 (%)		46.1/33.4/20.5	22.5/54.1/23.4	<0.001
Never consumed alcohol ages 16 to 43 (%)		1.7	1.4	0.897
Covariates				
Age 16	BMI at age 16 (kg/m^2^)	20.0.(2.6)	19.8 (2.6)	0.366
	BMI >25 at age 16 (%)	4.5	4.8	0.803
	Daily intake of sugary foods at age 16 (%)	17.3	14.9	0.339
	Daily smoking (%)	33.1	18.0	<0.001
	Exercise a few times a month or more seldom at age 16 (%)	21.0	26.3	0.060
	Family history of diabetes at age 16 (%)	3.3	3.5	0.835
	Both parents working-class at age 16 (%)	37.0	36.6	0.982
Age 43	BMI at age 43 (kg/m^2^)	25.8 (5.0)	27.5 (4.2)	<0.001
	BMI >25 at age 43 (%)	46.3	69.3	<0.001
	Daily intake of sugary foods at age 43 (%)	8.1	5.8	0.187
	Daily smoking at age 43 (%)	19.9	14.4	0.029
	Exercise a few times a month or more seldom at age 43 (%)	35.6	45.2	0.003
	Hypertension at age 43 (%)	16.8	32.3	<0.001

Data are displayed as mean (SD) for fasting plasma glucose and BMI, median (interquartile range, IQR) for alcohol consumption variables, and percentages (%),*p < .05

^a^Binge drinking (consuming four or more standard drinks (five for men) of alcohol on the same occasion at least once a month) from ages 16 to 43 (range 0–28) is defined as number of years during which the participant reported binge drinking

^b^Combined total alcohol consumption and binge drinking from ages 16 to 43 ranged from 0 (not binge drinking or alcohol consumption under 75th percentile ages 16–43) to 2 (binge drinking 1–28 years and alcohol consumption over 75th percentile)

A larger proportion of those who reported binge drinking at age 16 still engaged in that behavior at age 18 as compared to those who did not report binge drinking at age 16. This relationship was accentuated for women until age 21, but continued for men throughout all follow-ups (Table 2), which indicate that consumption patterns set in adolescence may be persistent later in life.

**Table 2:** Frequency and proportion of respondents who continue binge drinking at age 18, 20, 30, and 43 after reporting binge drinking at each preceding follow-up at ages 16, 18, 21, and 30

	Women	Men
	Binge drinking at age 18	Binge drinking at age 18
Binge drinking at age 16 n women = 94, n men = 87	48 (51.1%)	73 (83.9%)
	Binge drinking at age 21	Binge drinking at age 21
Binge drinking at age 18 n women = 92, n men = 160	41 (44.6%)	127 (79.4%)
	Binge drinking at age 30	Binge drinking at age 30
Binge drinking at age 21 n women = 83, n men = 253	33 (39.8%)	151 (59.7%)
	Binge drinking at age 43	Binge drinking at age 43
Binge drinking at age 30 n women = 93, n men = 222	30 (32.3%)	129 (58.1%)

Data displayed as frequencies (n) and proportions (%, row percent)

Table 3 showed significant differences in fPG levels between different categories of alcohol consumption for women, but not for men. There was a significant linear trend in the ANOVA analyses for women with regards to total alcohol consumption between ages 16 and 43 (*p*-value girls = 0.001, *p*-value boys = 0.65) and with regards to binge drinking between ages 16 and 43 (*p*-value girls = <0.001, *p*-value boys = 0.904). The largest fPG increase was found in the top quintiles of alcohol consumption. The quadratic term for the combined total consumption/binge drinking variable was however not significant.

**Table 3:** Mean (SD) fasting plasma glucose level according to quintiles of total alcohol consumption (ages 16 to 43), each quintile of binge drinking (ages 16 to 43), and each category of the combined total alcohol consumption and binge drinking variable

	Women		Men	
	Fasting plasma glucose age 43	p-value^a^	Fasting plasma glucose age 43	p-value^a^
Total alcohol consumption age 16 to 43				
1st quintile	4.81 (0.51)	0.002	5.37 (1.63)	0.355
2nd quintile	4.91 (0.57)		5.24 (0.85)	
3rd quintile	4.86 (0.54)		5.19 (0.75)	
4th quintile	4.89 (0.58)		5.23 (0.73)	
5th quintile	5.17 (0.83)		5.46 (0.79)	
Binge drinking age 16 to 43				
1st quintile	4.83 (0.50)	<0.001	5.30 (1.38)	0.904
2nd quintile	4.83 (0.50)		5.23 (0.92)	
3rd quintile	4.83 (0.68)		5.38 (0.97)	
4th quintile	4.84 (0.58)		5.27 (0.89)	
5th quintile	5.13 (0.69)		5.27 (0.52)	
Combined total alcohol consumption and binge drinking variable between age 16 and 43				
Zero periods of binge drinking and total alcohol consumption below 75th percentile	4.84 (0.52)	<0.001	5.31 (1.43)	0.470
Either binge drinking 1–5 periods or total alcohol consumption above 75th percentile	4.89 (0.59)		5.25 (0.89)	
Binge drinking 1–5 periods and total alcohol consumption above 75th percentile	5.19 (0.82)		5.39 (0.75)	

^a^Significance tested through ANOVA, *p < .05

Scatter plots on fPG and total alcohol consumption between ages 16 and 43 showed R^2^ = 0.015 for women and R^2^ = 0.002 for men, while the residual plot showed R^2^ = 0 for both women and men, indicating possible linear relationship the two variables. In addition, a multivariable linear regression analysis showed an insignificant quadratic trend (*p*-value = 0.134, VIF = 2.28) for the total alcohol consumption (ages 16 to 43) and fPG association.

Table 4 shows differences in dichotomized covariates with regards to fPG, total alcohol consumption and binge drinking (ages 16 to 43). Those who reported BMI <25, irregular exercise habits and hypertension at age 43 also had higher levels of fPG, total alcohol consumption, and binge drinking (ages 16 to 43). The patterns were similar between the two alcohol consumption variables.

**Table 4:** Characteristics of covariates by fasting plasma glucose (fPG) at age 43, total alcohol consumption ages 16 to 43, and binge drinking ages 16 to 43

	Fasting plasma glucose age 43^a^	*p*-value	Total alcohol consumption ages 16 to 43^b^	*p*-value	Binge drinking ages 16 to 43^c^	*p*-value
BMI >25 at age 16	5.35 (1.22)	0.061	28,237 (37937)	0.527	6.0 (17.5)	0.587
BMI <25 at age 16	5.11 (0.84)		30,177 (48981)		2.5 (18.0)	
Daily intake of sugary foods at age 16	5.07 (0.63)	0.891	39,010 (66483)	0.054	8.5 (20.0)	(0.225)
Less than daily intake of sugary foods at age 16	5.13 (0.90)		29,107 (44740)		6.0 (17.5)	
Daily smoking at age 16	5.09 (0.73)	0.442	**39,114 (61835)**	**<0.001**	**9.5 (21.0)**	**<0.001**
Less than daily smoking at age 16	5.13 (0.90)		**27,782 (44641)**		**6.0 (16.5)**	
Exercise a few times a month or more seldom at age 16	**5.19 (1.27)**	**0.015**	**42,284 (68154)**	**<0.001**	**11.0 (20.0)**	**<0.001**
Exercise at least once a week at age 16	**5.09 (0.68)**		**27,568 (43624)**		**6.0 (15.4)**	
Family history of diabetes at age 16	**5.41 (1.48)**	**0.020**	42,686 (58414)	0.853	7.0 (17.0)	0.774
No family history of diabetes at age 16	**5.11 (0.83)**		29,762 (47346)		6.0 (17.5)	
Both parents working-class at age 16	5.13 (0.72)	0.877	29,883 (47686)	0.833	**8.5 (17.8)**	**0.007**
No or one parent working-class at age 16	5.11 (0.93)		30,223 (50758)		**6.0 (17.5)**	
BMI >25 at age 43	**5.28 (1.00)**	**0.003**	**35,099 (56201)**	**0.005**	**8.5 (18.5)**	**<0.001**
BMI <25 at age 43	**4.88 (0.51)**		**26,523 (39977)**		**2.5 (11.5)**	
Daily intake of sugary foods at age 43	4.82 (0.58)	0.590	23,624 (41323)	0.229	4.8 (11.9)	0.286
Less than daily intake of sugary foods at age 43	5.14 (0.87)		30,386 (50030)		6.0 (17.5)	
Daily smoking at age 43	5.23 (0.72)	0.878	38,906 (68784)	0.071	**10.0 (20.0)**	**<0.001**
Less than daily smoking at age 43	5.10 (0.88)		28,725 (44546)		**6.0 (15.5)**	
Exercise a few times a month or more seldom at age 43	**5.21 (1.07)**	**0.013**	**35,643 (69638)**	**0.039**	**9.5 (20.0)**	**<0.001**
Exercise more than a few times a moth at age 43	**5.06 (0.67)**		**28,217 (39657)**		**6.0 (14.0)**	
Hypertensive at age 43	**5.45 (1.24)**	**0.001**	**41,110 (68651)**	**<0.001**	**9.5 (21.0)**	**0.001**
Normotensive at age 43	**5.00 (0.64)**		**27,576 (43291)**		**6.0 (15.5)**	

Data are displayed as mean (SD) for (which is normally distributed), and as median (IQR) for total alcohol consumption ages 16 to 43 and binge drinking ages 16 to 43 (which are not normally distributed). Significant differences in bold, *p < .05

^a^Mmol/L. ^b^Grams of pure alcohol. ^c^Binge drinking from ages 16 to 43 (range 0–28) is defined as number of years during which the participant reported binge drinking

In the univariate linear regression analyses, BMI, smoking, and hypertension at age 43 were associated with fPG at age 43 (Table 5) for women. In addition, fPG was univariately associated with all three alcohol consumption variables (total alcohol consumption, binge drinking, and the combined binge drinking-total alcohol consumption variable) for women. This was in contrast to men where only hypertension at age 43, BMI and exercise at age 16 and 43 were associated with fPG.

**Table 5:** Univariate linear regressions with fasting plasma glucose at age 43 as the dependent variable by gender

	Univariate analyses					
		Women			Men	
	Unst. Beta (CI)	St. Beta	*p*-value	Unst. Beta (CI)	St. Beta	*p*-value
Total alcohol consumption from ages 16 to 43	**0.000000229 (0.00–0.00)**	**0.180**	**<0.001**	0.0000000249 (0.00–0.00)	0.054	0.249
Binge drinking from ages 16 to 43^a^	**0.019 (0.01–0.03)**	**0.222**	**<0.001**	−0.001 (−0.01–0.01)	−0.008	0.859
Combination of binge drinking and total alcohol consumption from ages 16 to 43^b^	**0.110 (0.06–0.16)**	**0.204**	**<0.001**	0.043 (−0.05–0.13)	0.030	0.526
BMI at age 16	0.009 (−0.02–0.03)	0.035	0.465	**0.055 (0.02–0.09)**	**0.146**	**0.002**
Intake of sugary foods at age 16	−0.010 (−0.07–0.05)	−0.017	0.724	0.009 (−0.08–0.10)	0.009	0.844
Smoking at age 16	0.033 (−0.02–0.09)	0.059	0.222	0.02 (−0.08–0.12)	0.019	0.690
Exercise at age 16	−0.029 (−0.07–0.02)	−0.062	0.203	**0.069 (0.01–0.13)**	**0.106**	**0.023**
Family history of diabetes at age 16	0.17 (−0.16–0.50)	0.048	0.318	0.412 (−0.09–0.91)	0.076	0.107
Working-class parents at age 16	−0.013 (−0.09–0.06)	−0.017	0.719	0.074 (−00.04–0.19)	0.060	0.198
BMI at age 43	**0.035 (0.02–0.05)**	**0.277**	**<0.001**	**0.072 (0.05–0.09)**	**0.305**	**<0.001**
Intake of sugary foods at age 43	−0.044 (−0.11–0.02)	−0.066	0.173	−0.051 (−0.16–0.05)	−0.044	0.343
Smoking at age 43	**0.106 (0.05–0.16)**	**0.180**	**<0.001**	0.061 (−0.03–0.15)	0.061	0.196
Exercise at age 43	0.015 (−0.02–0.05)	0.041	0.397	**0.061 (0.01–0.12)**	**0.104**	**0.026**
Hypertension at age 43	**0.218** **(0.07–0.37)**	**0.138**	**0.004**	**0.484** **(0.29–0.68)**	**0.226**	**<0.001**

Data are displayed as unstandardized beta (confidence interval), standardized beta, *p*-value. Significant results are in bold, *p < .05

^a^Binge drinking (consuming four or more standard drinks (five for men) of alcohol on the same occasion at least once a month) from ages 16 to 43 (range 0–28) is defined as number of years during which the participant reported binge drinking

^b^Combined total alcohol consumption and binge drinking from ages 16 to 43 ranged from 0 (not binge drinking or alcohol consumption under 75th percentile ages 16 to 43) to 2 (binge drinking 1–28 years and alcohol consumption over 75th percentile)

In the multivariable analyses, the total alcohol consumption variable (adjusted R^2^ = 0.112), the binge drinking variable (adjusted R^2^ = 0.107) as well as the combination of binge drinking and total alcohol consumption variable (adjusted R^2^ = 0.111) remained significantly associated with fPG among women, even after adjusting for BMI, hypertension and smoking at age 43 (Table 6). For men, only BMI and hypertension at age 43 remained associated with fPG in the multivariable analysis (adjusted R^2^ = 0.118).

**Table 6:** Multivariable linear regressions with fasting plasma glucose at age 43 as the dependent variable. Women only

	Multivariable model 1^c^			Multivariable model 2^d^			Multivariable model 3^e^		
	Women			Women			Women		
	Unst. Beta (CI)	St. Beta	*p*-value	Unst. Beta (CI)	St. Beta	*p-*value	Unst. Beta (CI)	St. Beta	*p-*value
Total alcohol consumption from ages 16 to 43	**0.000000689** **0.00–0.00**	**0.138**	**0.004**						
Binge drinking from ages 16 to 43^a^				**0.01** **0.002–0.02**	**0.121**	**0.012**			
Combination of binge drinking and total alcohol consumption from ages 16 to 43^b^							**0.11** **0.03–0.13**	**0.144**	**0.003**

Data are displayed as unstandardized beta (confidence interval), standardized beta, *p*-value. Significant results are in bold, *p < .05

^a^Binge drinking (consuming four or more standard drinks (five for men) of alcohol on the same occasion at least once a month) from ages 16 to 43 (range 0–28) is defined as number of years during which the participant reported binge drinking

^b^Combined total alcohol consumption and binge drinking from ages 16 to 43 ranged from 0 (not binge drinking or alcohol consumption under 75th percentile ages 16 to 43) to 2 (binge drinking 1–28 years and alcohol consumption over 75th percentile)

^c^Model 1 included total alcohol consumption from ages 16 to 43, BMI, smoking, and hypertension at age 43

^d^Model 2 included binge drinking from ages 16 to 43, BMI, smoking, and hypertension at age 43

^e^Model 3 included the combined binge drinking and total alcohol consumption variable, BMI, smoking, and hypertension at age 43

We tested other cut-offs in the multivariable linear regressions for the combined binge drinking and total alcohol consumption variable, but the association remained (data not shown). Stratifying for BMI at age 43 to examine the possible role of BMI as an effect modifier did not change the results significantly (data not shown). When added to the multivariable regressions, waist circumference at age 43 as well as weight change between ages 16 and 43 showed similar association as for BMI at age 43 (data not shown). Moreover, since family history of diabetes, weight change, and working class are known risk factors for diabetes, all three variables were inserted as confounders in the same multivariate regression analysis; however, this did not change the results in a significant way (data not shown). Finally, excluding five possible total alcohol consumption outliers and never-consumers did not significantly alter the results.

## Discussion

In this unique study with 27 years of follow-up, we found that higher alcohol consumption, together with binge drinking behavior, was associated with high fasting plasma glucose in adult women. The association was found in women independent of their BMI, smoking behavior, or them being hypertensive. These findings are in contrast to some previous studies in adults that showed that women who were moderate alcohol consumers (~24 g/d) had a lower risk for developing type 2 diabetes [1, 2]. However, as noted in a recent meta-analysis, women consuming large amounts (>50 g/d) of alcohol had an increased risk of diabetes [2].

Furthermore, our results did not show that being overweight/obese modified the relationship between alcohol consumption and fPG, which also stands in contrast to previous studies [1, 21]. There are several possible explanations for the discrepancies between our results and those of previous studies. First, previous studies were performed in adults, where other conditions such as long-term obesity or liver disease could confound the association between alcohol consumption and fPG. Our sample was followed from youth through middle age with the same questionnaire and only a few had developed a severe drinking problem that could have resulted in severe liver disease. Secondly, most of the previous studies have used different categories of consumers as reference category, and if a study cannot separate abstainers from former drinkers, this could confound the association and contribute to the U-shape of the association curve in their logistic models [1]. In contrast, we relied on continuous variables as both the dependent (fPG) and independent (total alcohol consumption) variables thereby avoiding the reference category problem. Thirdly, it is difficult to compare our measure of total alcohol consumption with the cross sectional data of consumption per day as in previous reports.

There are some mechanistic studies that might explain the association. Hyperinsulinemic-euglycemic clamp studies in men have shown that ethanol increased insulin resistance by competing with the oxidation of e.g. carbohydrates [22]. Moreover, it has been shown in rats that binge drinking increased hypothalamic inflammation and expression of protein tyrosine phosphatase 1B (PTP1B) which is a negative regulator of insulin signaling [23].

Differences in associations between higher alcohol consumption and elevated glucose levels between men and women are difficult to explain; however, the difference in effect of alcohol has been noted in several other studies [1, 3] but none of these studies have been able to identify any mechanistic explanations to the observed gender difference. Thus, more experimental studies are needed.

### Strengths and limitations

The methodological strengths of the present study were its design consisting of a prospective cohort study spanning over 27 years and its very high retention rate. The question about selection bias must always be addressed. The cohort consists of all pupils in the last year of compulsory school in a whole municipality in Northern Sweden and has, during the long-term follow-up, retained a very high response rate. The cohort has also proved to be representative of the corresponding age group in Sweden with regard to demographic, health-related and socioeconomic factors [10, 13]. Thus, the selection bias in this study is low. However, in a closed cohort such as this, the cohort is more ethnically homogenous compared to Sweden today [10]. Another methodological strength is that we analyzed three types of alcohol consumption measures, thereby covering both consumption levels and patterns, which has been shown as important by previous research [24].

The measure of total alcohol consumption and binge drinking between ages 16 and 43 were determined using the trapezium rule in an area under the curve calculation, which has the advantage including duration of time in longitudinal studies. This approach has been recommended in serial measurement studies [25, 26]. These are approximations because we have no information about participants’ drinking habits between the follow-ups. However, prospective studies are highly desirable for lifetime measures of alcohol consumption as compared with retrospective studies [25, 27]. The questionnaire items on alcohol consumption were phrased e.g. ‘How often do you drink wine?’ and ‘How much wine do you usually drink on each occasion?’. As in all self-reported survey items, there is a general risk of recall bias when the cohort participants were asked to report on their alcohol consumption. A test-retest investigation was performed at baseline, in which the results from four of the 46 included school classes in Northern Swedish Cohort was compared with a study two weeks later that used exactly the same alcohol questionnaire. Overall, the comparison showed high degree of conformity between the two studies.

Using a continuous measure also has the advantage of not reducing variation in the data due to categorization. In our regression analyses, we tried to avoid this by using not collapsing our continuous or ordinal scale variables into different categories. The disadvantage of measuring the cumulative volume in alcohol consumption is that we run the risk of losing information about the consumption patterns, i.e. different consumption patterns may result in the same volume consumed. We have attempted to avoid this by adding the binge drinking measurement. The definition of binge drinking comprised the quantity of the alcohol consumed per occasion within a certain time-frame (at least once a month), and taking gender into account. This way of defining binge drinking was suggested in a review of binge drinking measurements in epidemiological studies [17]. The review also suggested to include a third variable, time-frame of consumption (e.g. within two hours); however, we did not have access to such data.Future studies are important to elaborate on the importance of alcohol consumption patterns in the population.

When creating the alcohol consumption variables, we used the concept of a ‘standard drink’, defined by the Public Health Agency of Sweden as containing 12 g of alcohol [18]. The amount of alcohol in a standard drink differs somewhat between countries [18, 28], which is important to consider when making international comparisons.

Although the results in Model 3 (Table 6) might be interpreted as binge drinking having an additional and increased risk in relation to fPG beyond that of total alcohol consumption, we cannot make such conclusions. Due to limitations in our sample size, we are not able to make certain claims of the independent role of binge drinking. Moreover, we cannot assess the exact mechanism behind the observed association because we do not have any information on liver function, gluconeogenesis, insulin resistance, etc. Also, although self-reports of alcohol consumption are considered rather valid, it is important to consider tendencies either under- or overestimate self-reports [29]. Linear regressions were performed when possible outliers were excluded but the results were practically the same. Another limitation of our study was that we did not have access to data on fPG at age 16, polycystic ovary syndrome or data on gestational diabetes at age 43.

## Conclusion

In conclusion, our study shows that high alcohol consumption ages 16–43 was associated with increased fasting plasma glucose for non-diabetic women. A raised blood glucose concentration is the significantly most important risk factor for future development of type 2 diabetes. Thus, our findings indicate that reducing binge drinking and alcohol consumption among young and middle-aged women with the highest consumption might be of benefit for reducing their risk of developing type 2 diabetes with its long term complications.

The public health relevance of this paper is that early interventions directed towards prevention of adolescent alcohol consumption are important for prevention of the development of type 2 diabetes in adulthood. Our findings have a broader implication for public health as alcohol consumption in adolescence can be regarded as an externalizing symptom of mental ill health [30, 31]. Thus, our study indicates the long-term public health consequences of early mental ill health. Future research is needed about the possible mechanisms from a life-course perspective.

## Abbreviations

ANOVA: Analysis of variance
BMI: Body Mass Index
EQUALIS: External quality assurance in laboratory medicine in Sweden
fPG: Fasting plasma glucose
IQR: Interquartile range
SD: Standard deviations
WHO: World Health Organisation
WHO MONICA: World Health Organisation Multinational Monitoring of Trends and Determinants of Cardiovascular Disease
